# Testing the limits: the impact of extreme functional hip width on the muscular demands of walking

**DOI:** 10.1242/bio.062747

**Published:** 2026-07-17

**Authors:** Cristina Gildee, Patricia Ann Kramer

**Affiliations:** ^1^ Department of Anthropology, University of Washington, Seattle, WA, USA; ^2^ Department of Orthopaedics, University of Washington, Seattle, WA, USA

**Keywords:** Obstetrical dilemma, Pelvic width, Musculoskeletal modeling, Bipedal locomotion, Muscle metabolic energetics

## Abstract

The obstetrical dilemma (OD) posits that hominin pelvic morphology reflects a trade-off between obstetric demands and efficient bipedal locomotion – wider pelves facilitate childbirth but increase mechanical demands on stabilizing muscles, potentially raising the energetic cost of walking. Several studies challenge this view, however, demonstrating no strong associations between pelvic width and increased energy expenditure, suggesting that musculoskeletal compensation minimizes locomotor costs via muscle coordination and posture dynamics. Because assessment of evolutionary constraints requires examination of forms outside observed species variation, we used musculoskeletal simulations to evaluate how extreme deviations in functional hip width affect gait parameters. Motion capture and ground reaction data from ten adults (45 trials) drove subject-specific musculoskeletal models where functional hip widths were altered by ±20% and ±40%. Simulations used identical gait kinematics to isolate the effect of pelvic breadth. Wider pelves increased hip abductor force and metabolic power during mid-stance, while narrower pelves showed inverse trends. Compensatory changes in antagonistic muscle groups (e.g. adductors and flexors) limited net effects. Across all pelvic conditions, the dominant contributors to stride and muscle metabolic cost remained consistent, and total muscle metabolic power stayed within observed inter-individual variation. These results support hypotheses suggesting human musculoskeletal systems can adapt to extreme shifts in pelvic width without substantial muscle metabolic penalties. Contrary to core assumptions of the OD, pelvic breadth alone does not constrain walking effectiveness, suggesting that pelvic evolution was shaped primarily by reproductive, developmental, and structural pressures than by locomotor energetics alone.

## Teaser

**Summary:** Musculoskeletal simulation of extreme pelvic widths shows that compensatory muscle redistribution keeps total metabolic cost within normal variation, suggesting pelvic breadth alone does not constrain walking.

## INTRODUCTION

The relationship between pelvic morphology and bipedal locomotor efficiency has long been explored through the lens of the obstetrical dilemma (OD). Initially proposed by Washburn (1960), the OD posits that human pelvic shape represents an evolutionary constraint and subsequent trade-off between two competing demands: facilitating childbirth and maintaining efficient bipedal locomotion (Washburn, 1960). Specifically, the OD hypothesis argues that the requirement for a wider pelvic inlet to accommodate the passage of large-brained neonates conflicts with biomechanical advantages associated with a narrower pelvis that reduces the energetic cost of stabilizing the pelvis during upright walking (Washburn, 1960; Ruff, 1991; Rosenberg, 1992).

The biomechanical principle underlying this dilemma is that a broader pelvis increases the lateral distance between hip joints, lengthening the moment arm for the body's weight during the single-leg stance phase of bipedal gait. This increased moment arm theoretically generates greater coronal-plane moments about the hip, thereby necessitating increased muscle forces, particularly from hip abductors, to stabilize the pelvis and resist pelvic drop when one foot is off the ground (Ruff, 1991; Rosenberg, 1992). Thus, wider pelves are assumed to incur greater metabolic cost during walking due to increased muscular demand.

Recent empirical studies, however, provide robust evidence challenging this traditional view. Warrener et al. (2015) found no significant relationship between pelvic width and whole-body energy expenditure in modern human populations, finding that wider pelves do not inherently compromise locomotor efficiency (Warrener et al., 2015). Wall-Scheffler and Myers (2017) demonstrated potential biomechanical advantages of wider pelves in women, such as increased effective step length and improved balance. Their results provide a more nuanced interpretation, whereby the musculoskeletal system's adaptability may mitigate energetic penalties that broader pelvic morphology might theoretically impose.

Musculoskeletal modeling has further advanced our understanding of these biomechanical interactions. In previous work, Kramer and Sylvester (2023, 2025a) employed computational modeling to show that variations within typical human functional hip widths resulted in negligible differences in muscle metabolic energetics during walking. Their results indicate that although increased functional hip width is associated with higher coronal-plane hip moments, this does not translate directly into increased muscle metabolic energy expenditures (Kramer and Sylvester, 2023, 2025a); additionally, they indicate that previous biomechanical models utilizing single, static, coronal-plane snapshots of pelvic forces and moments are insufficient to accurately establish the real-world muscle energetic requirements to support pelvic stability during hominin locomotion. Further, humans likely adjust their gait and muscle coordination patterns in subtle yet effective ways to maintain locomotor efficiency despite anatomical differences.

The relationship between pelvic morphology and locomotor efficiency is particularly significant in the context of early hominins. *Australopithecus afarensis*, exemplified by the fossil specimen ‘Lucy’ (A.L. 288-1), is believed to have had a considerably broader pelvis relative to stature than anatomically modern humans (Tague and Lovejoy, 1986; Lovejoy, 1988; Häusler and Schmid, 1995). This morphology has traditionally been interpreted as energetically disadvantageous for efficient walking (Ruff, 1991). Recent modeling studies (Kramer and Sylvester, 2025a; Stansfield et al., 2026) have, however, demonstrated that even pelvic morphologies scaled well beyond documented modern human variation could achieve efficient bipedal gait with moderate redistributions of muscular load rather than the presumed substantial increases in overall muscular energy expenditure.

Given the absence of clear energetic costs within the natural range of human pelvic variation, it remains unknown how significantly pelvic dimensions must deviate from typical human forms before producing meaningful differences in locomotor energetics. Empirically testing such extremes is impractical due to biological constraints. Computational musculoskeletal modeling, however, provides an innovative approach to exploring these hypothetical morphological extremes. By artificially manipulating the functional hip width (i.e. the distance between the left and right hip joint centers of rotation), models can systematically evaluate the biomechanical and energetic consequences of pelvic dimensions that are substantially wider or narrower than those found in extant human populations (Kramer and Sylvester, 2023, 2025b; Stansfield et al., 2026).

Building upon this methodological foundation, this study aims to systematically investigate the hypothetical consequences of substantial deviations in human pelvic width through musculoskeletal simulation. This approach is a physics-based experiment and not an attempt to replicate biologically effective individuals. That is, the extremes that we produce are unlikely to be biologically viable. Specifically, we have manipulated functional hip width by altering the distance between hip joint centers by ±20% and ±40% beyond typical human variation. By maintaining consistent walking kinematics across these conditions, we isolate the effect of pelvic morphology on muscle force and metabolic energy expenditure.

We hypothesize, in alignment with traditional interpretations of the OD hypothesis, that significantly wider pelves will increase hip abductor activation and consequently elevate overall muscle force and metabolic costs during the stance phase of walking. Conversely, narrower pelvic configurations are predicted to reduce these metrics. Given the interconnected nature of the musculoskeletal system and the hip joint's integral role in the lower-limb kinetic chain, we also anticipate systemic compensatory effects (e.g. muscle forces might be redistributed across multiple muscle groups). That is, some muscle groups may experience increased activation, while others decrease their activity, ultimately moderating any net energetic impact (Kramer and Sylvester, 2025b). By exploring pelvic morphologies outside of modern human variation, we aim to investigate the biomechanical and energetic implications of extreme pelvic dimensions, providing valuable insights into the broader constraints shaping the evolution of hominin pelvic morphology.

## RESULTS

**Fig. 1.**
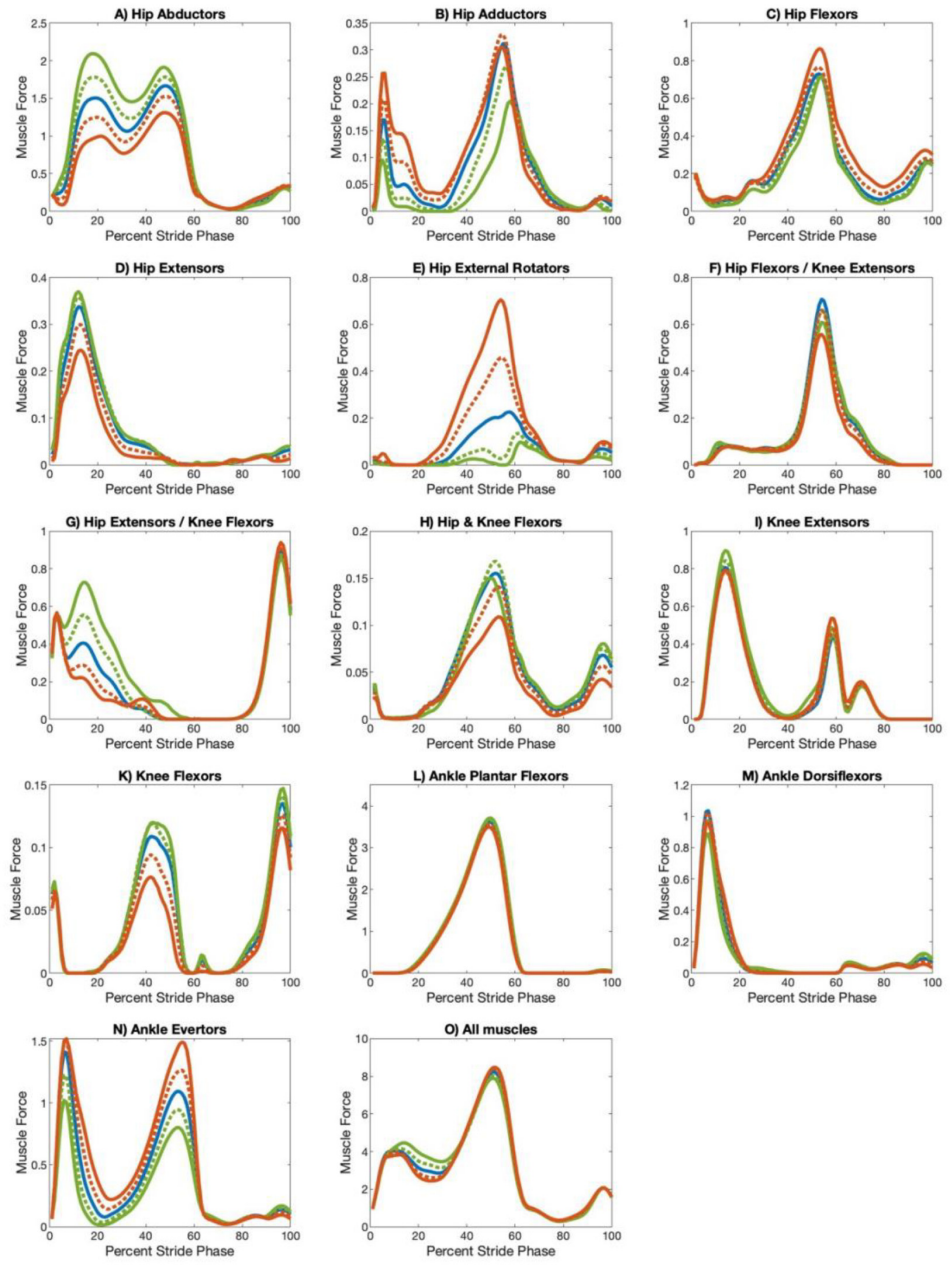
**Normalized muscle functional group forces (in multiples of body weight) for a stride.** (A−O) The blue solid line is the original functional pelvic width. Green lines are for increased functional pelvic width, while orange lines are for decreased functional pelvic width; solid lines are 40% changes, while dashed lines are 20% changes. Each subfigure is for a muscle functional group or, in the case of O, the sum of all muscle functional groups. 0% stride is heel strike, and 100% is the next heel strike of the ipsilateral foot.

**Fig. 2.**
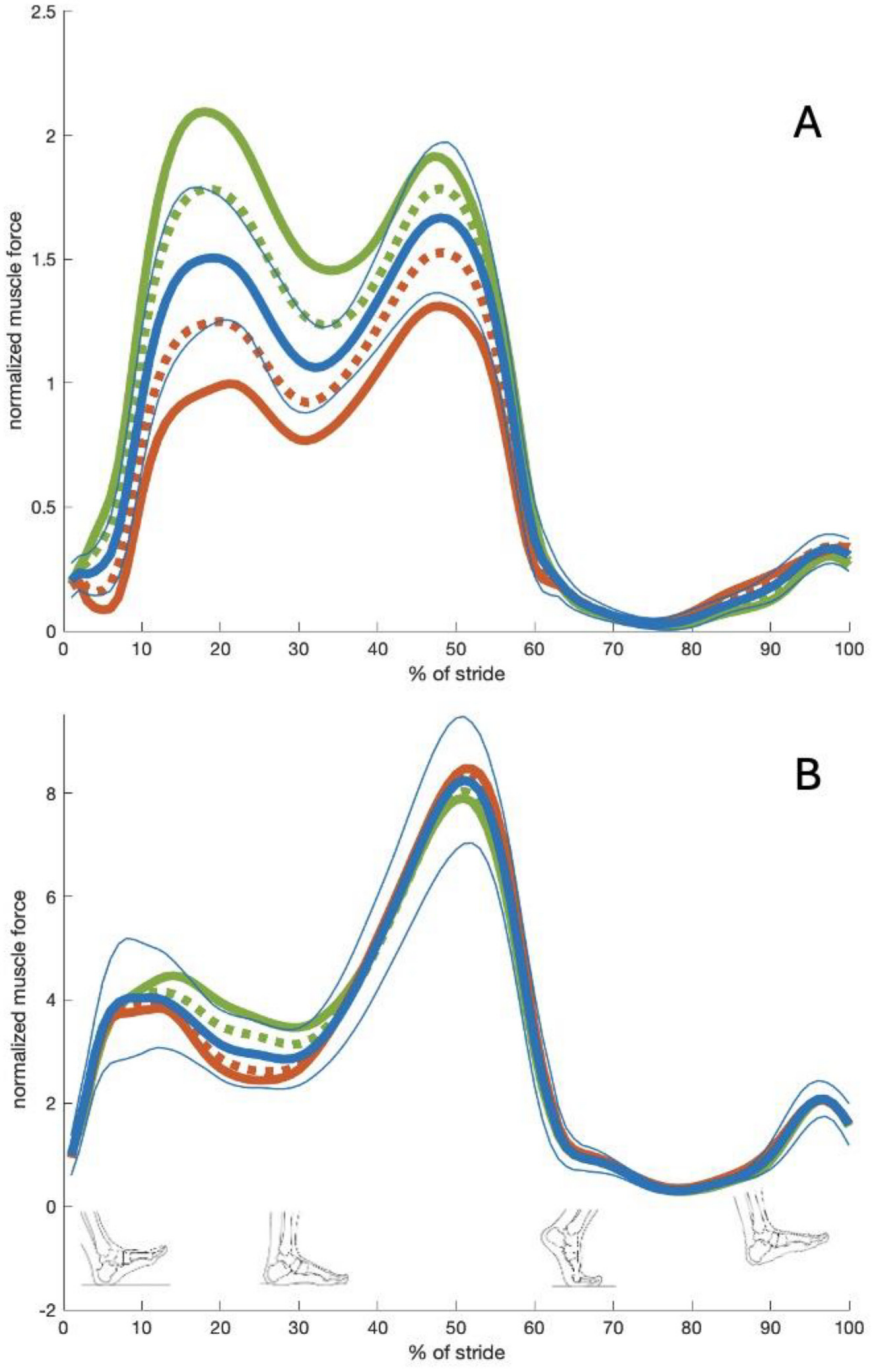
**Normalized hip abductor (A) and total (B) muscle forces (in multiples of body weight) for a stride.** Blue solid lines are the original functional pelvic width; the thick line is the average, while the thin lines are ±1 s.d. Green lines are for increased functional pelvic width, while orange lines are for decreased functional pelvic width; solid lines are 40% changes, while dashed lines are 20% changes. 0% stride is heel strike, and 100% is the next heel strike of the ipsilateral foot.

Manipulations of functional hip width produced modest, compensatory shifts in muscle force or power but did not substantially alter total muscle forces or energetic cost predictions (Fig. 1; Fig. S1). Statistical parametric mapping (SPM) showed significant differences between baseline and the width-adjusted conditions in most muscle functional groups and across most of the gait cycle (Fig. S2). Specifically for the hip abductors, wider pelves (increased by 20% and 40% beyond typical human variation) demonstrated elevated muscle force and power use in the single-stance phase, as expected (Fig. 2A). This increase, however, fell within the range of inter-individual variability in total muscular force (Fig. 2B).

The hip, hip-knee, and knee extensor groups also demonstrated higher muscle force with wider pelves; however, wider pelvis conditions exhibited reduced muscle force and metabolic power in the hip adductors, flexors, and external rotators. This pattern of increased requirements in some muscle groups but decreased in others suggests a complex compensatory redistribution of muscular effort between antagonistic muscle groups. The narrower pelvis configurations showed elevated metabolic activity in the hip adductors, flexors, and external rotators, as well as ankle evertors and dorsiflexors, indicating altered stabilization demands.

Across all pelvic width conditions, dominant contributors to total stride muscle force and power (namely, the plantar flexors) displayed consistent patterns in both timing and magnitude and were substantially unaffected by pelvic morphology adjustments. Overall, the experimental manipulations did not push total muscle metabolic power beyond the natural inter-individual variability observed in the typical pelvis condition. Rather, the results indicate a redistribution of muscle force and metabolic energy use within the musculoskeletal system.

### Limitations

Our simulations maintained gait kinematics across all pelvic configurations to isolate the effect of a change in width and did not allow kinematic change. We acknowledge that it is likely that if an individual had a wider or narrower pelvis, they might adapt their gait (e.g. altering step width or length, trunk sway, or cadence) to maintain comfort and balance; such gait adaptations could further mitigate any energetic impact. For instance, a person with a very wide pelvis might naturally walk with a slightly longer step length (Rak, 1991; Gruss et al., 2017). We did not allow this compensation, effectively testing an extreme, albeit limited pelvic morphology. Even under these extreme width conditions, the fact that whole-body differences among widths appear to be similar to those among individuals suggests that the system is robust. Future studies could explore optimizing gait adjustments in concert with anatomical changes.

Additionally, our metabolic energy calculations were model-based estimations relying on assumed muscle efficiency constants. While such methods effectively identify relative energetic differences, they cannot precisely replicate subtle physiological nuances like muscle-fiber-type variability. Other models of muscle metabolic energy use [e.g. those of Umberger et al. (2003) and Bhargava et al. (2004)] are potentially more representative of the physiological parameters; however, they rely on muscle parameters that are difficult to acquire. The Margaria (1968) approach simply uses efficiency parameters. While this approach is unlikely to yield accurate values of muscle metabolic power, comparison among models using the same approach is informative. Nonetheless, our results depend on the relative accuracy of the efficiency parameters and their relationship. More accuracy in musculoskeletal models depends on the development of more physiologically accurate muscle models, but future musculoskeletal work might address the sensitivity of models to the efficiency parameters.

Lastly, our study highlights the integrated nature of musculoskeletal responses, with functional hip width adjustments influencing multiple interconnected muscle groups. Consequently, we acknowledge that single-factor correlations between pelvic width and locomotor energetics may oversimplify complex biomechanical relationships, emphasizing the need for comprehensive, multifaceted modeling approaches in future evolutionary and functional studies of pelvic morphology.

## DISCUSSION

Our results provide a detailed biomechanical evaluation of how substantial deviations in pelvic width affect locomotor energetics, supporting our hypothesis that significant alterations in pelvic width are accommodated through coordinated muscular adjustments, which maintain locomotor effectiveness without incurring meaningful energetic penalties. This result challenges key elements of Washburn's OD. The traditional OD hypothesis posits a significant energetic penalty for wider pelvic dimensions, driven by increased hip abductor muscle activation and force necessary for stabilizing the pelvis during bipedal gait (Washburn, 1960; Ruff, 1991; Rosenberg, 1992). Our results partially support this perspective, as we observed increases in hip abductor force and metabolic power when pelvic width was increased substantially (20–40%); however, this elevation in metabolic power is within observed inter-individual variability, suggesting that pelvic width alone does not impose a significant metabolic penalty on walking efficiency.

As predicted, wider pelvic configurations led to compensatory reductions in muscle activation or force production elsewhere, notably in the hip adductors, indicating a redistribution of muscular workload across antagonistic muscle groups. This biomechanical trade-off reflects the integrated nature of the musculoskeletal system, demonstrating that the bipedal body could efficiently mitigate potential energetic costs via subtle shifts in muscle coordination (Kramer and Sylvester, 2023, 2025b). These results align closely with previous work demonstrating no clear relationship between pelvic width and locomotor energetics in modern populations (Warrener et al., 2015; Wall-Scheffler and Myers, 2017), supporting interpretations of the OD that complicate its more traditional pelvic width limitation (Fischer and Mitteroecker, 2017; Fischer et al., 2021; Haeusler et al., 2021; Stansfield et al., 2021a,b; Grunstra et al., 2023).

This pattern is consistent with that of Stansfield et al. (2026), who used predictive forward dynamics modeling to scale pelvic width by up to ±50% and likewise found that whole-body metabolic cost rose only marginally (∼1% for a ±20% width change), with lower-limb length exerting a substantially larger effect. Notably, however, they observed that abductor-specific energy demands increased in proportion to pelvic width even as whole-body cost remained stable, leading them to propose that any locomotor constraint on pelvic width acted at the subsystem level (i.e. through muscle-specific energetics and fatigue) rather than through whole-body energy economy. Our results align with this interpretation and support their conclusion that pelvic evolution was unlikely to have been constrained by whole-body locomotor economy alone.

Our results also extend the conclusions of prior work demonstrating the minimal effects of pelvic morphology variations on muscle energetics within typical human ranges (Kramer and Sylvester, 2023). Even when modeling hypothetical extremes of functional hip width beyond observed human variation, the musculoskeletal system appears robustly adaptable, adjusting muscle activation patterns to maintain overall energetic effectiveness. This adaptability could explain why broader pelvic morphologies found in certain early hominins, such as *A. afarensis* (Lovejoy, 1988; Häusler and Schmid, 1995), may not have imposed significant energetic disadvantages. Indeed, previous modeling suggests that even the considerably wider australopithecine-like pelvis could hypothetically facilitate efficient bipedalism without incurring prohibitive energy costs through similar compensatory adjustments in muscular load (Kramer and Sylvester, 2025a).

The lack of substantial energetic penalties observed here challenges simplistic interpretations of the OD that emphasize a strict biomechanical compromise. Instead, our results align with recent evolutionary anthropological frameworks suggesting that pelvic morphology evolution in hominins has been shaped by multiple overlapping selective pressures, not solely walking locomotor efficiency (Fischer and Mitteroecker, 2015, 2017; Fischer et al., 2021; Haeusler et al., 2021; Stansfield et al., 2021a,b, 2026), and contribute to the broader discussion regarding pelvic evolution and parturition biomechanics. While pelvic dimensions have traditionally been considered overwhelmingly constrained by obstetric requirements, our results suggest substantial flexibility in the pelvic musculoskeletal system, capable of accommodating morphological extremes with little effect on whole-body locomotor energetic costs. Our results support arguments emphasizing the potential evolutionary significance of factors other than energetic efficiency, such as pelvic floor stability, thermoregulation, and developmental plasticity, as primary drivers of pelvic form (Wells et al., 2012; Stansfield et al., 2021a,b).

### Conclusion

This study provides compelling evidence that significant deviations in pelvic width do not substantially compromise locomotor effectiveness, even when exaggerated beyond typical human variation. Rather, the musculoskeletal system exhibits considerable adaptability, efficiently redistributing muscular loads to maintain metabolic costs during bipedal walking. Given that varying human anatomy *in vivo* is impossible, musculoskeletal simulations offer the opportunity to examine many of these anatomical questions *in silico*. Our results challenge the traditional energetic constraints proposed by the OD, suggesting that a broader set of selective pressures beyond locomotor energetics influenced pelvic evolution. Future investigations should continue exploring the multifaceted interactions between pelvic morphology, obstetric demands, and other evolutionary pressures such as pelvic floor integrity, developmental constraints, and thermoregulatory factors to fully understand the diversity observed in human pelvic morphology.

## Ethics

This study is a secondary computational analysis of a publicly available, openly licensed (CC BY 4.0) gait dataset (Schreiber and Moissenet, 2019); no new human-subjects data were collected. Ethical approval for the original data collection was granted by the Joint Institutional Ethics Committee of the Rehazenter (Luxembourg) in accordance with the Declaration of Helsinki, and all participants provided informed consent, as reported by the original authors. Secondary analysis of these de-identified data did not require additional institutional review.

## MATERIALS AND METHODS

### Experimental data and model parameterization

We utilized an existing dataset of human gait collected by Schreiber and Moissenet (2019), which provides motion capture and ground reaction force data for 50 adults walking at various speeds. From this dataset, we selected ten healthy adults (five females and five males) to encompass a broad range of body sizes (stature and mass). Each subject walked at their self-selected comfortable speed over a walkway with floor-embedded force plates, completing multiple trials. We selected the five available trials of straight-level walking at the participant's preferred walking velocity (50 total trials), of which 45 were suitable for analysis. A minimum of three trials per participant were included in simulations. Key anthropometric measurements (e.g. stature, body mass, and segment lengths) for these individuals are given in Table 1 (see supplementary information for complete data).

**Table 1.** Anthropometric characteristics of subjects

Subject ID	Gender	Age (years)	Stature (m)	Mass (kg)	Functional hip width (m)		
					Original	+40%	−40%
2014001	M	31	1.66	67	0.176	0.246	0.105
2014013	W	26	1.70	61	0.158	0.221	0.095
2014014	M	29	1.80	92	0.178	0.249	0.107
2014016	W	46	1.69	76	0.194	0.271	0.116
2014019	W	26	1.76	74	0.171	0.240	0.103
2014031	M	21	1.77	67	0.165	0.231	0.099
2014040	W	19	1.55	57	0.159	0.223	0.095
2014048	W	40	1.64	62	0.171	0.240	0.103
2014051	M	25	1.91	88	0.191	0.268	0.115
2015030	M	24	1.87	86	0.192	0.268	0.115

From Schreiber and Moissenet (2019), except for functional hip width (original and variations), which is the distance between hip joint centers as determined through parameters.

Gait event timing (heel-strike and toe-off) was identified, kinematic data were recorded, and ground reaction forces were measured via a 52-marker full-body marker set and ground-embedded force plates, respectively, as previously described to allow for precise stride segmentation (Schreiber and Moissenet, 2019). The kinematics and kinetics from one complete stride (initial contact of one foot to the subsequent ipsilateral initial contact) were extracted for each trial and used as inputs for musculoskeletal modeling.

We employed the AnyBody Modeling System (version 7.4, AnyBody Technology, Denmark) to create subject-size-specific musculoskeletal models. As a base, we used the freely available ADL_Gait model (beta version), representing a complete anatomically modern human musculoskeletal system for gait. This model includes nine rigid segments in the lower body (pelvis, left and right femora, tibiae, tali, and feet) plus additional segments for the trunk, head, and arms. The lower body musculature is represented by 169 muscle-tendon elements per leg, based on the Twente Lower Extremity Model 2.1 dataset (Carbone et al., 2015; De Pieri et al., 2018). Each subject's model was obtained by scaling this generic model to match their anthropometry and then calibrating joint and marker driver locations via a process called parameterization (or in AnyBody ParameterIdentification).

First, we input each subject's stature, body mass, and segment length measurements into the AnyBody scaling tool, which uniformly scales segment dimensions to approximate the individual's size. Then, a more detailed optimization is performed to fine-tune segment lengths and joint center locations using that subject's dynamic motion data. Because internal joint centers (e.g. hip joint centers) cannot be directly measured without invasive medical imaging, the model's parameter identification algorithm adjusts those joint parameters to best fit the experimentally observed marker trajectories during the dynamic trials.

A critical aspect of model calibration is the alignment of the pelvis segment. We imposed an anatomical constraint defining neutral pelvic tilt: the anterior pelvic plane (APP), defined by the two anterior superior iliac spines (ASIS) and the pubic tubercles, was set to within ±5° of vertical (parallel to the coronal plane) when the subject stood in a static pose. This criterion follows previous recommendations (Mayr et al., 2005; Marques et al., 2018) to achieve a consistent ‘neutral’ reference orientation for different pelves. To this end, we allowed the model's virtual ASIS markers to be slightly adjusted relative to experimental markers until the APP angle was within 5° of vertical on average during the stride (with <5° deviation at mid-stance). Pelvic tilt optimization was necessary because variations in pelvic morphology can cause standard marker placements to yield apparent tilt differences in the APP by >20° between individuals (Preece et al., 2008). By aligning each model's pelvis to a common reference (approximately 0–5° tilt) during standing, we ensured that differences in pelvic shape did not introduce large initial pose discrepancies (Brown-Taylor et al., 2020).

The parameterization algorithm also handles most marker pairing optimizations automatically; however, this requires that certain virtual markers (e.g. greater trochanter or lateral femoral epicondyle) must be fixed to the experimental marker data to ‘ground’ the model during optimization (Sylvester et al., 2021a). This ensured, for instance, that the virtual pelvis did not drift away from the subject's recorded pelvis position. Through these procedures, each subject-specific model was obtained with personalized segment dimensions (for both overall size and segment proportions) while retaining the generic model's musculoskeletal structure (Fig. S3). Functional hip width is the distance between the hip joint centers after parametrization (Table 1).

After parameterization, each subject's dynamic data were applied to drive an inverse dynamics simulation in AnyBody. The parameterized marker driver trajectories were used to impose segment and joint motions, and the recorded ground reaction forces were applied at the foot segment contacts. AnyBody solves the dynamic equilibrium equations directly from segment motions. We used the built-in muscle redundancy solver, which distributes loads among the muscle elements by minimizing the sum of the instantaneous muscle activations cubed (i.e. an optimization criterion with a power of 3) (Andersen, 2021). This polynomial criterion yields a physiologically realistic recruitment pattern that neither overemphasizes nor underutilizes any one muscle group (Kramer et al., 2022). In this context, muscle activation is a unitless value that is the ratio of instantaneous muscle force divided by the maximum voluntary contraction strength.

### Metabolic power estimation calculations

In addition to instantaneous muscle element forces, AnyBody also provides instantaneous muscle contraction/extension velocity from segment motion and mechanical power from muscle force and acceleration. An estimate of muscle metabolic power is calculated assuming that the gross metabolic muscle power is approximated as P_met_=P_mech_/η, where P_met_ is the metabolic power estimate, P_mech_ is the mechanical power, and η is an efficiency factor (assumed ∼0.25 for positive and 1.25 for negative muscle mechanical powers) (Margaria, 1968). The outputs of the inverse dynamic simulations for each subject's original model included time-varying activations, forces, and metabolic powers for all 169 muscle elements per leg.

### Pelvic width manipulation

We conducted a set of simulations altering the functional hip width of the models. Starting from each subject's original scaled model, we increased and decreased the distance between the left and right hip joint centers by 20% and 40%. This was achieved by rescaling the pelvis segment in the mediolateral direction while leaving the legs and other body segments unaltered. A 40% increase in hip breadth was chosen to approximate the extreme ratio seen in *A. afarensis*. For context, the ‘Lucy’ pelvis has been estimated to be about 30–35% wider relative to femur length than a human of equivalent stature (Jungers, 1982; Tague and Lovejoy, 1986). We slightly exaggerated this to 40% to confidently encompass that range. Likewise, a 40% reduction produced an extraordinarily narrow pelvis that far exceeds normal human variation in the opposite direction. We also included ±20% changes as intermediate conditions to observe scaling trends (i.e. whether muscle effects scale linearly or nonlinearly with width). These values represent hypothetical extremes rather than realistic modern human variation, consistent with our aim of exploring the boundary at which pelvic width constrains locomotion.

For each adjusted model, the hip joint centers were repositioned laterally (outward for widening, inward for narrowing) by the specified percentage of the original functional hip width (see Table 1 for resulting values). Importantly, we did not manually alter the subjects' gait kinematics in tandem with these anatomic changes. Each widened or narrowed model was driven through the same gait motions (joint angles and stride parameters) as the original, unaltered model. This isolates the effect of pelvic width itself, rather than confounding it with potential gait adjustments. We did not change stride width for two reasons. First, changing stride width changes kinematic patterns, which we did not intend to do. Second, the stride width for australopithecines is within the normal human range (Tuttle, 1987), even though the australopithecine pelvis is broad relative to femoral or overall leg length.

We recognize that an individual with a very wide or very narrow pelvis might adopt a different gait (e.g. changes in step width, step length, trunk lean, and knee flexion) to accommodate their anatomy. Our experimental design intentionally fixes the kinematics so that differences in outcome measures can be attributed solely to the change in pelvic geometry. In our view, this is an extreme scenario for a wide or narrow pelvis because we do not allow the minor gait adjustments that almost certainly would accrue with a different pelvic anatomy.

All other model parameters (e.g. body mass, segment lengths, and muscle properties) remained the same as in the subject's baseline model for these width experiments. The only difference was the mediolateral scaling of the pelvis segment and corresponding reposition of the hip joint and muscle attachments. Each of the ±20% and ±40% pelvis models underwent the same inverse dynamics and muscle force algorithm calculations described above, using the subject's original kinematic and kinetic data as input.

### Simulation outputs and calculations

We grouped the 169 muscle elements into anatomical muscles (e.g. all elements representing gluteus medius summed into one curve) and into muscle functional groups for easier interpretation as previously described (Sylvester et al., 2021b). Muscle force magnitudes were summed as a measure of the demand on the musculoskeletal system; that is, the muscle ‘force’ of the anatomical muscles or muscle functional groups (e.g. hip adductors or knee flexors) is not the resultant of all muscle element forces, but rather the sum of all muscle element force magnitudes. For each model (±20% width, ±40% width), we obtained time-varying muscle functional group forces and estimated muscle metabolic power curves for all muscle functional groups over a complete gait cycle.

### Statistical analysis

We applied one-dimensional SPM to test for differences in muscle functional group force and metabolic power between the experimental conditions. Because our outcome variables are continuous waveforms spanning the gait cycle rather than discrete scalars, SPM was used to test the entire time series while controlling for multiple comparisons; this avoids the loss of temporal information and inflated error that arise when a single point or peak is used for analysis.

For each muscle functional group, we conducted a repeated-measures analysis of variance (ANOVA) across the time-normalized gait cycle (101 points from 0% to 100% stance). The factor was hip configuration (original compared to +20% width, +40% width, −20% width, −40% width), and subjects were treated as a random repeated factor.

To accommodate the balanced-design requirement of SPM ANOVA, we included three trials per subject per condition; for subjects with more than three trials, we randomly selected three. The SPM{F} statistic was calculated as a function of time over the gait cycle (Pataky et al, 2013). A result was considered significant if the SPM{F} curve crossed the critical threshold (corresponding to α=0.05) for any contiguous time interval. All statistical analyses were performed in MATLAB with the spm1d package (v0.4). We report whether and when in the gait cycle significant differences were found, which provides insight into both the magnitude and the timing of any effects (e.g. whether differences manifest during mid-stance versus late stance).

## Supplementary Material

10.1242/biolopen.062747_sup1 Supplementary information

## References

[BIO062747C1] Andersen, M. S. (2021). Introduction to musculoskeletal modelling. In *Computational modelling of biomechanics and biotribology in the musculoskeletal system*, pp. 41-80. Woodhead Publishing. 10.1016/B978-0-12-819531-4.00004-3

[BIO062747C2] Bhargava, L. J., Pandy, M. G. and Anderson, F. C. (2004). A phenomenological model for estimating metabolic energy consumption in muscle contraction. *J. Biomech.* 37, 81-88. 10.1016/S0021-9290(03)00239-2 14672571

[BIO062747C3] Brown-Taylor, L., Schroeder, B., Lewis, C. L., Perry, J., Hewett, T. E., Ryan, J. and Di Stasi, S. (2020). Sex-specific sagittal and frontal plane gait mechanics in persons post-hip arthroscopy for femoroacetabular impingement syndrome. *J. Orthop. Res.* 38, 2443-2453. 10.1002/jor.24680 32249962 PMC7541416

[BIO062747C4] Carbone, V., Fluit, R., Pellikaan, P., van der Krogt, M. M., Janssen, D., Damsgaard, M., Vigneron, L., Feilkas, T., Koopman, H. F. J. M. and Verdonschot, N. (2015). TLEM 2.0 – a comprehensive musculoskeletal geometry dataset for subject-specific modeling of lower extremity. *J. Biomech.* 48, 734-741. 10.1016/j.jbiomech.2014.12.034 25627871

[BIO062747C5] De Pieri, E., Lund, M. E., Gopalakrishnan, A., Rasmussen, K. P., Lunn, D. E. and Ferguson, S. J. (2018). Refining muscle geometry and wrapping in the TLEM 2 model for improved hip contact force prediction. *PLoS ONE* 13, e0204109. 10.1371/JOURNAL.PONE.0204109 30222777 PMC6141086

[BIO062747C6] Fischer, B. and Mitteroecker, P. (2015). Covariation between human pelvis shape, stature, and head size alleviates the obstetric dilemma. *Proc. Natl. Acad. Sci. USA* 112, 5655-5660. 10.1073/pnas.1420325112 25902498 PMC4426453

[BIO062747C7] Fischer, B., Grunstra, N. D. S., Zaffarini, E. and Mitteroecker, P. (2021). Sex differences in the pelvis did not evolve de novo in modern humans. *Nat. Ecol. Evol.* 5, 625-630. 10.1038/s41559-021-01425-z 33767411 PMC7617076

[BIO062747C8] Fischer, B. and Mitteroecker, P. (2017). Allometry and sexual dimorphism in the human pelvis. *Anat. Rec.* 300, 698-705. 10.1002/ar.23549 28297185

[BIO062747C9] Grunstra, N. D. S., Betti, L., Fischer, B., Haeusler, M., Pavlicev, M., Stansfield, E., Trevathan, W., Webb, N. M., Wells, J. C. K., Rosenberg, K. R. et al. (2023). There is an obstetrical dilemma: misconceptions about the evolution of human childbirth and pelvic form. *Am. J. Biol. Anthropol.* 181, 535-544. 10.1002/ajpa.24802 37353889 PMC10952510

[BIO062747C10] Gruss, L. T., Gruss, R. and Schmitt, D. (2017). Pelvic breadth and locomotor kinematics in human evolution. *Anat. Rec.* 300, 739-751. 10.1002/ar.23550 PMC6560644 28297175

[BIO062747C11] Haeusler, M., Grunstra, N. D. S., Martin, R. D., Krenn, V. A., Fornai, C. and Webb, N. M. (2021). The obstetrical dilemma hypothesis: there's life in the old dog yet. *Biol. Rev.* 96, 2031-2057. 10.1111/brv.12744 34013651 PMC8518115

[BIO062747C12] Häusler, M. and Schmid, P. (1995). Comparison of the pelves of Sts 14 and AL288-1: implications for birth and sexual dimorphism in australopithecines. *J. Hum. Evol.* 29, 363-383. 10.1006/jhev.1995.1063

[BIO062747C13] Jungers, W. L. (1982). Lucy's limbs: skeletal allometry and locomotion in *Australopithecus afarensis*. *Nature* 297, 676-678. 10.1038/297676a0

[BIO062747C14] Kramer, P. A. and Sylvester, A. D. (2023). Hip width and metabolic energy expenditure of abductor muscles. *PLoS ONE* 18, e0284450. 10.1371/journal.pone.0284450 37071649 PMC10112780

[BIO062747C15] Kramer, P. A. and Sylvester, A. D. (2025a). Hip stabilization in an australopithecine-like hip: the influence of shape on muscle activation. *Biol. Open* 14, bio061931. 10.1242/BIO.061931/368195 40476334 PMC12208404

[BIO062747C16] Kramer, P. A. and Sylvester, A. D. (2025b). Peroration: hip width and metabolic energy expenditure of abductor muscles. *Am. J. Biol. Anthropol.* 187, e70075. 10.1002/AJPA.70075 40519191

[BIO062747C17] Kramer, P. A., Feuerriegel, E. M., Lautzenheiser, S. G. and Sylvester, A. D. (2022). Sensitivity of musculoskeletal models to variation in muscle architecture parameters. *Evol. Hum. Sci.* 4, e6. 10.1017/EHS.2022.6 37588892 PMC10426084

[BIO062747C18] Lovejoy, C. O. (1988). Evolution of human walking. *Sci. Am.* 259, 118-125. 10.1038/scientificamerican1188-118 3212438

[BIO062747C19] Margaria, R. (1968). Positive and negative work performances and their efficiencies in human locomotion. *Int. Z. Angew. Physiol.* 25, 339-351. 10.1007/BF00699624 5658204

[BIO062747C20] Marques, C. J., Martin, T., Kochman, A., Goral, A., Lampe, F., Breul, V. and Kozak, J. (2018). Pelvic tilt angle differences between symptom-free young subjects and elderly patients scheduled for THA: the rationale for tilt-adjusted acetabular cup implantation. *Open Orthop. J.* 12, 364-372. 10.2174/1874325001812010364 30288191 PMC6142662

[BIO062747C21] Mayr, E., Kessler, O., Prassl, A., Rachbauer, F., Krismer, M. and Nogler, M. (2005). The frontal pelvic plane provides a valid reference system for implantation of the acetabular cup: spatial orientation of the pelvis in different positions. *Acta Orthop.* 76, 848-853. 10.1080/17453670510045471 16470440

[BIO062747C22] Pataky, T. C., Robinson, M. A. and Vanrenterghem, J. (2013). Vector field statistical analysis of kinematic and force trajectories. *J. Biomech.* 46, 2394-2401. 10.1016/j.jbiomech.2013.07.031 23948374

[BIO062747C23] Preece, S. J., Willan, P., Nester, C. J., Graham-Smith, P., Herrington, L. and Bowker, P. (2008). Variation in pelvic morphology may prevent the identification of anterior pelvic tilt. *J. Man. Manip. Ther.* 16, 113-117. 10.1179/106698108790818459 19119397 PMC2565125

[BIO062747C24] Rak, Y. (1991). Lucy's pelvic anatomy: its role in bipedal gait. *J. Hum. Evol.* 20, 283-290. 10.1016/0047-2484(91)90011-J

[BIO062747C25] Rosenberg, K. R. (1992). The evolution of modern human childbirth. *Am. J. Phys. Anthropol.* 35, 89-124. 10.1002/ajpa.1330350605

[BIO062747C26] Ruff, C. B. (1991). Climate and body shape in hominid evolution. *J. Hum. Evol.* 21, 81-105. 10.1016/0047-2484(91)90001-C

[BIO062747C27] Schreiber, C. and Moissenet, F. (2019). A multimodal dataset of human gait at different walking speeds established on injury-free adult participants. *Sci. Data* 6, 111. 10.1038/s41597-019-0124-4 31270327 PMC6610108

[BIO062747C28] Stansfield, E., Kumar, K., Mitteroecker, P. and Grunstra, N. D. S. (2021a). Biomechanical trade-offs in the pelvic floor constrain the evolution of the human birth canal. *Proc. Natl. Acad. Sci. USA* 118, e2022159118. 10.1073/pnas.2022159118 33853947 PMC8072325

[BIO062747C29] Stansfield, E., Fischer, B., Grunstra, N. D. S., Pouca, M. V. and Mitteroecker, P. (2021b). The evolution of pelvic canal shape and rotational birth in humans. *BMC Biol.* 19, 224. 10.1186/S12915-021-01150-W 34635119 PMC8507337

[BIO062747C30] Stansfield, E., Egner, C., Mitteroecker, P. and Kainz, H. (2026). Did energy costs of walking limit the evolution of a larger human birth canal? *Proc. Biol. Sci.* 293, 20252895. 10.1098/rspb.2025.2895 41667102

[BIO062747C31] Sylvester, A. D., Lautzenheiser, S. G. and Kramer, P. A. (2021a). A review of musculoskeletal modelling of human locomotion. *Interface Focus* 11, 20200060. 10.1098/RSFS.2020.0060 34938430 PMC8361578

[BIO062747C32] Sylvester, A. D., Lautzenheiser, S. G. and Kramer, P. A. (2021b). Muscle forces and the demands of human walking. *Biol. Open* 10, bio058595. 10.1242/BIO.058595 34279576 PMC8325943

[BIO062747C33] Tague, R. G. and Lovejoy, C. O. (1986). The obstetric pelvis of A.L. 288-1 (Lucy). *J. Hum. Evol.* 15, 237-255. 10.1016/S0047-2484(86)80052-5

[BIO062747C34] Tuttle, R. H. (1987). Kinesiological inferences and evolutionary implications from laetoli bipedal trails G-1, G-2/3, and A. In *Laetoli: a Pliocene site in Northern Tanzania* (ed. M. D. Leakey and J. M. Harris), pp. 503-523. Oxford: Oxford University Press.

[BIO062747C35] Umberger, B. R., Gerritsen, K. G. M. and Martin, P. E. (2003). A model of human muscle energy expenditure. *Comput. Methods Biomech. Biomed. Engin.* 6, 99-111. 10.1080/1025584031000091678 12745424

[BIO062747C36] Wall-Scheffler, C. M. and Myers, M. J. (2017). The biomechanical and energetic advantages of a mediolaterally wide pelvis in women. *Anat. Rec.* 300, 764-775. 10.1002/ar.23553 28297181

[BIO062747C37] Warrener, A. G., Lewton, K. L., Pontzer, H. and Lieberman, D. E. (2015). A wider pelvis does not increase locomotor cost in humans, with implications for the evolution of childbirth. *PLoS ONE* 10, e0118903. 10.1371/JOURNAL.PONE.0118903 25760381 PMC4356512

[BIO062747C38] Washburn, S. L. (1960). Tools and human evolution. *Sci. Am.* 203, 62-75. 10.1038/scientificamerican0960-62 13843002

[BIO062747C39] Wells, J. C. K., DeSilva, J. M. and Stock, J. T. (2012). The obstetric dilemma: an ancient game of Russian roulette, or a variable dilemma sensitive to ecology? *Yearb. Phys. Anthropol.* 149, 40-71. 10.1002/ajpa.22160 23138755

