# Supplementary material for: Testing the limits: the impact of extreme functional hip width on the muscular demands of walking
Source: Biol Open. 2026 Jul 17;15(7):bio062747. doi: 10.1242/bio.062747 (PMC13446569; doi:10.1242/bio.062747)
Supplement: Supplementary file 1 [file biolopen-15-062747-s1.pdf]

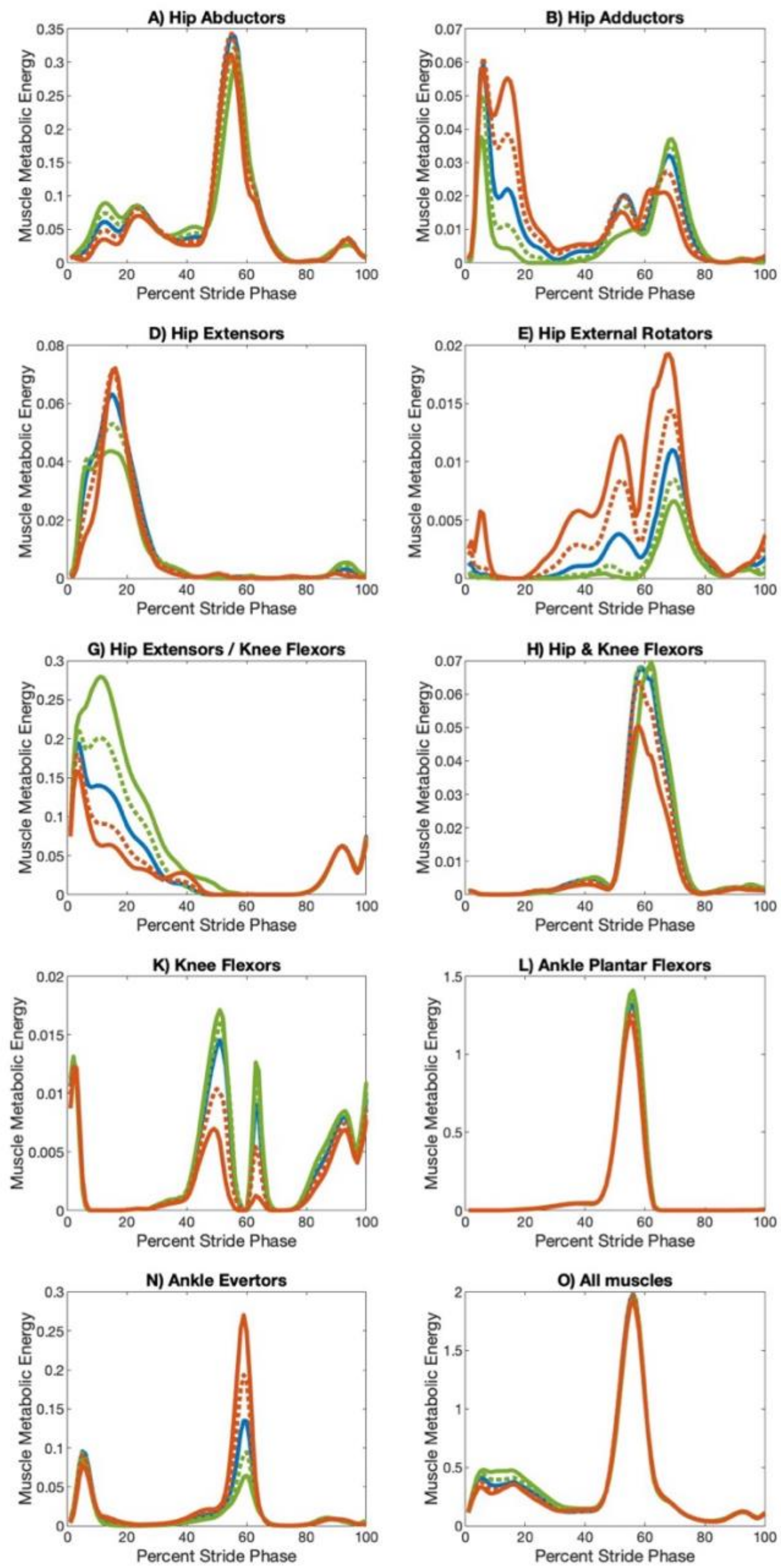

Fig. S1. Normalized muscle metabolic power for a stride. Blue solid line is the original functional pelvic width. Green lines are for increased functional pelvic width while orange lines are for decreased functional pelvic width; solid line are 40% changes while dashed lines are 20% changes. Each subfigure is for a muscle functional group or, in the case of O), the sum of all muscle functional groups. 0% stride is heel strike and 100% is the next heel strike of the ipsilateral foot.

Biology Open • Supplementary information

Muscle force, Wide 40%:

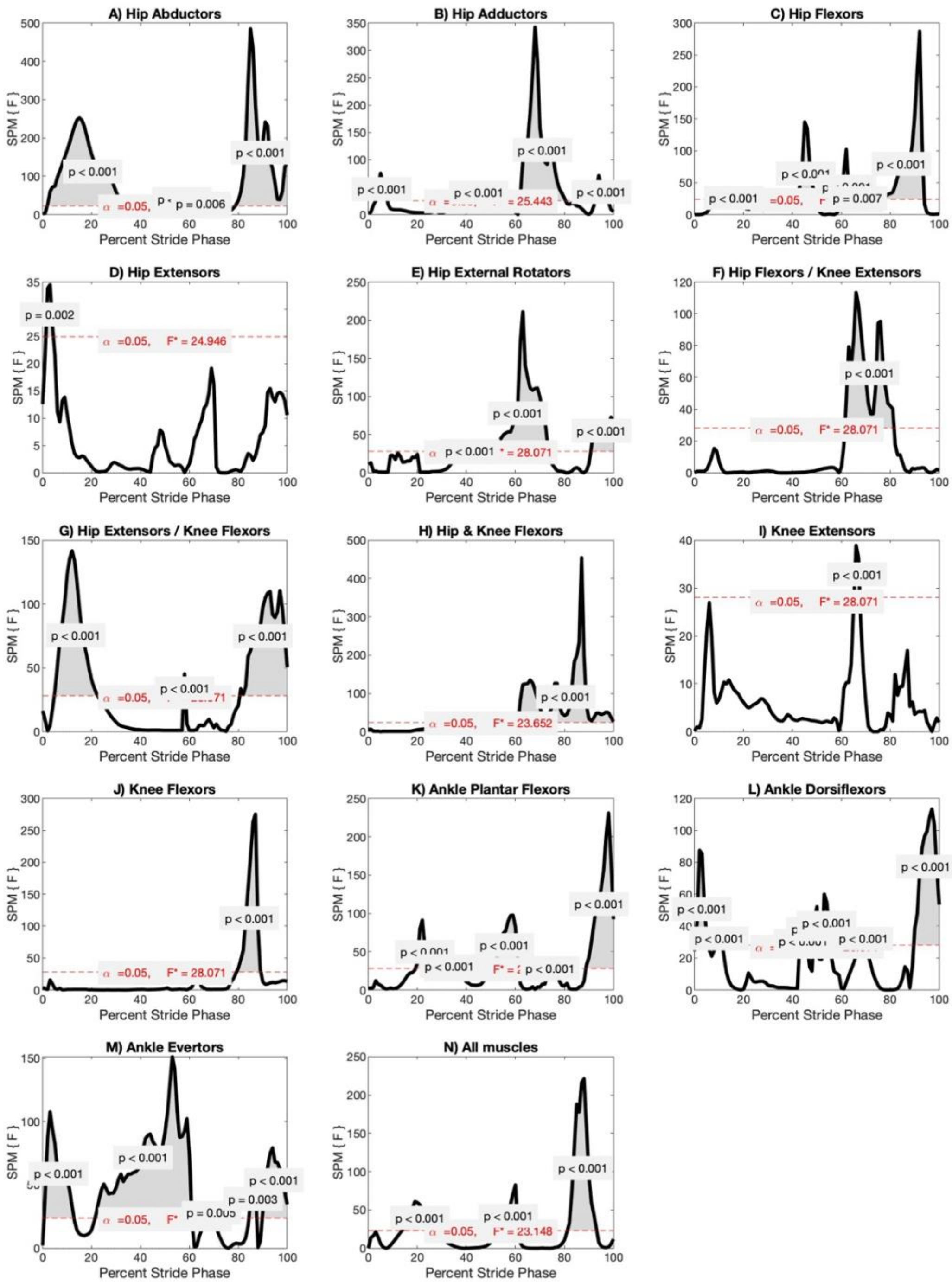

Muscle force, Wide 20%:

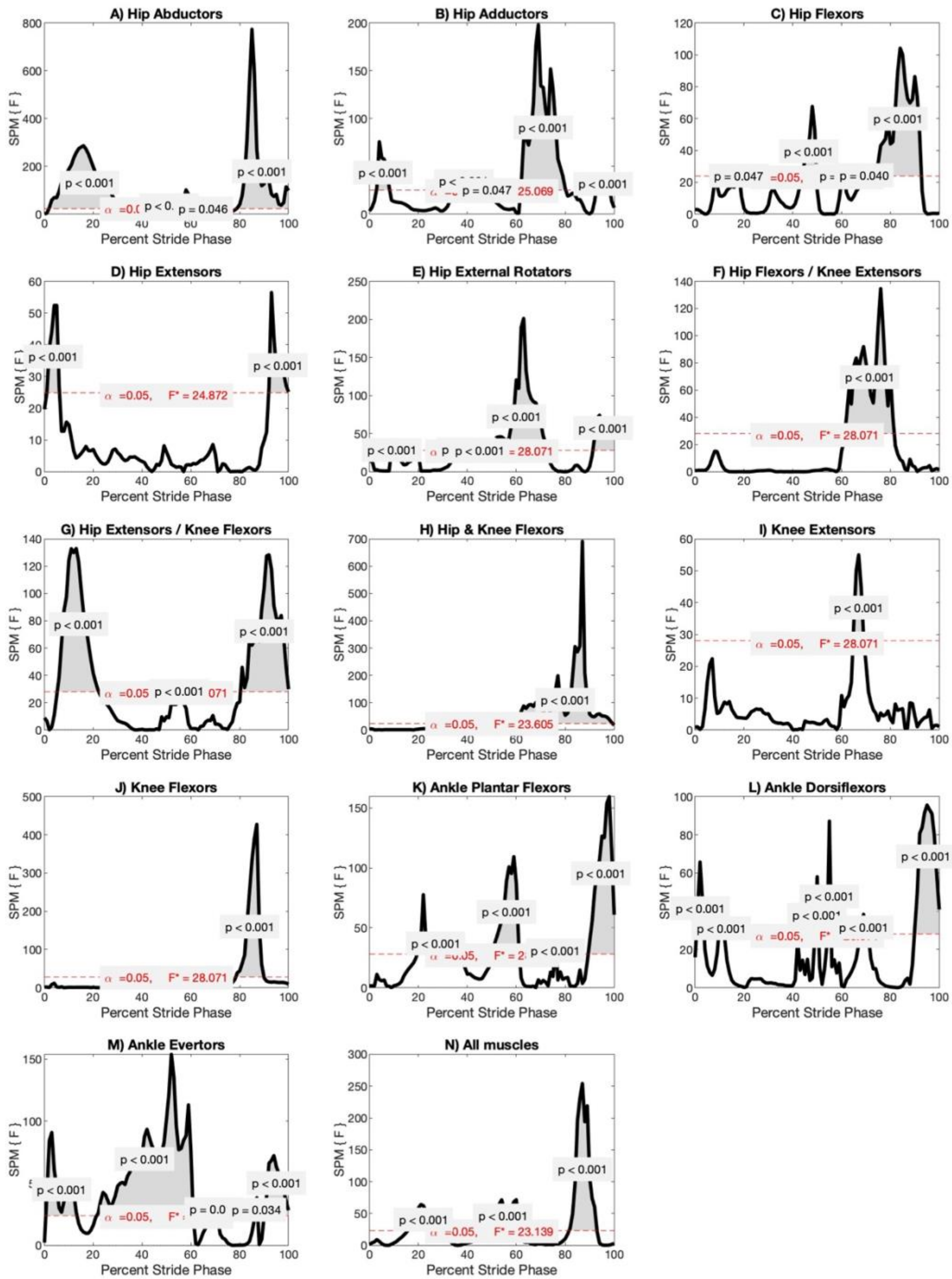

Biology Open • Supplementary information

Muscle force, Narrow 20%:

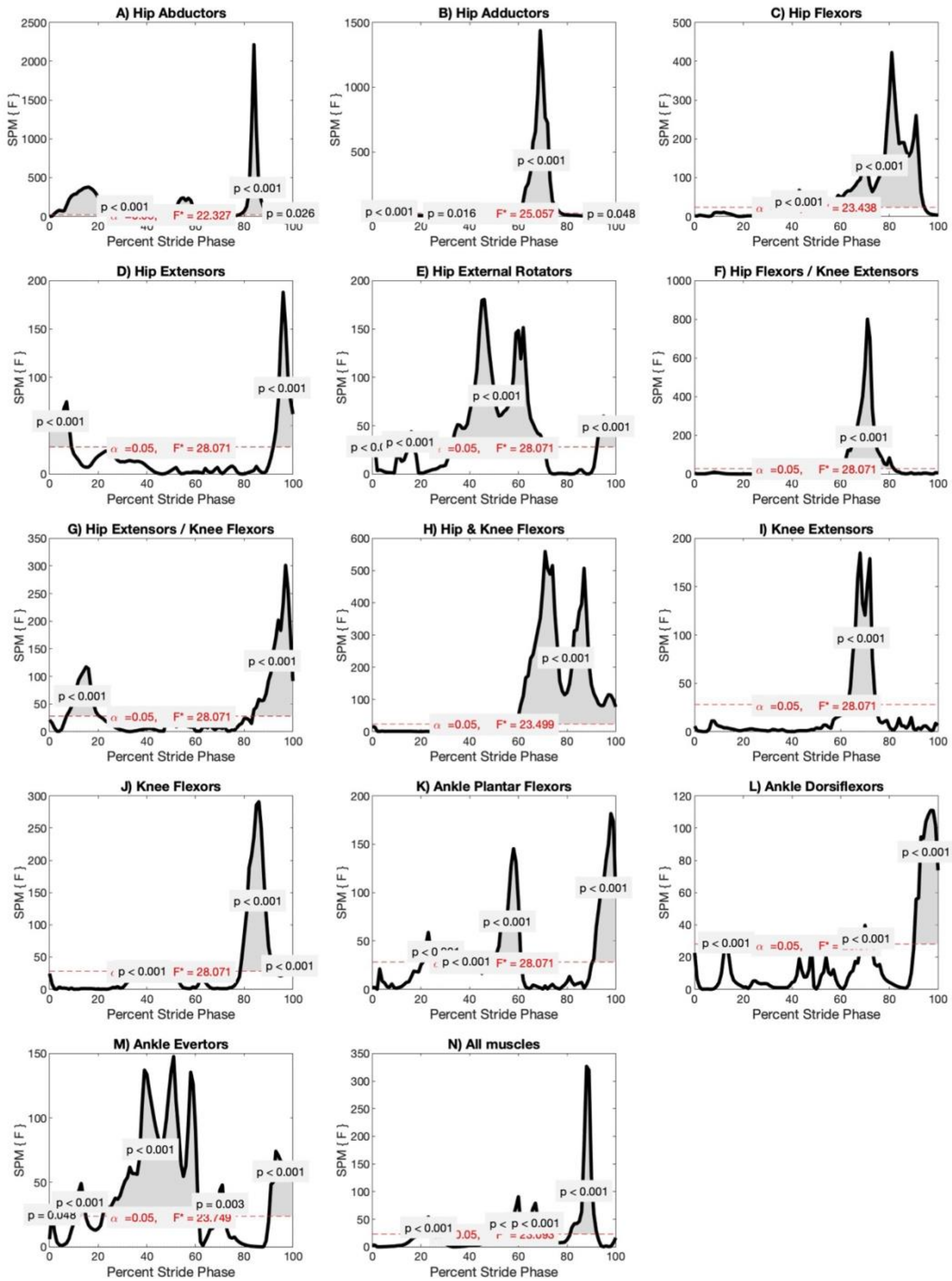

Biology Open • Supplementary information

Muscle force, Narrow 40%:

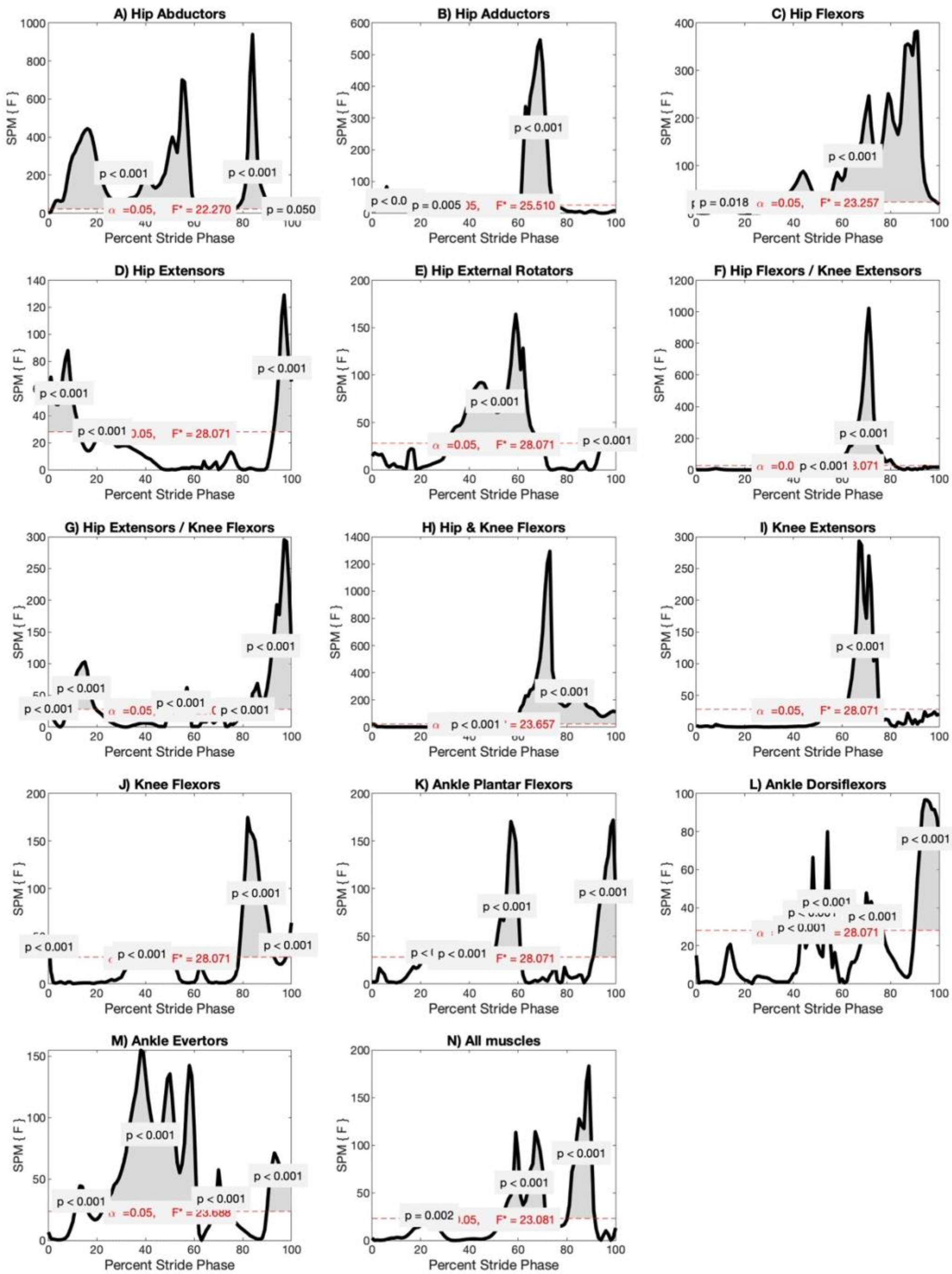

Biology Open • Supplementary information

Muscle metabolic power, Wide 40%:

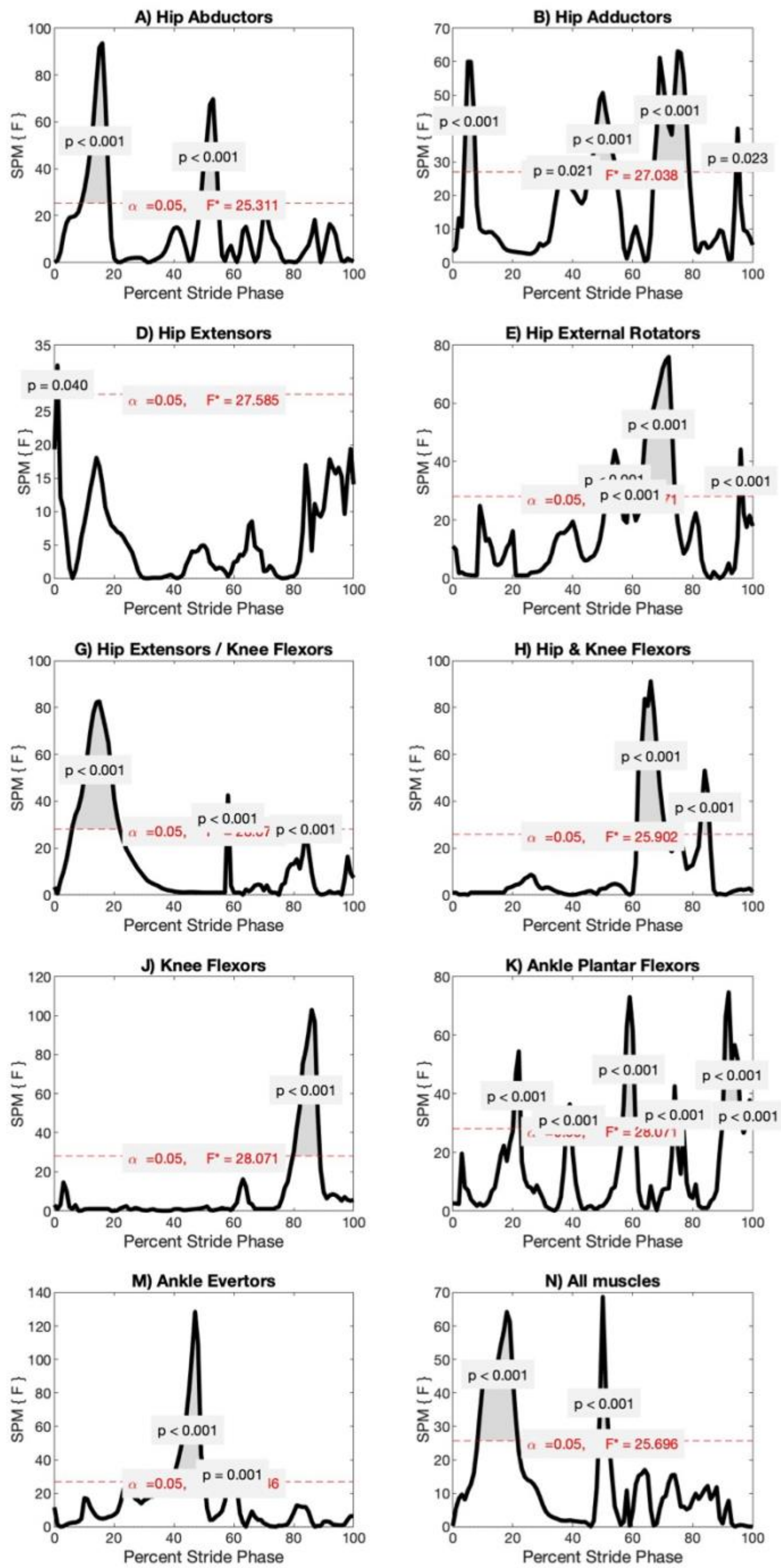

Biology Open • Supplementary information

Muscle metabolic power, Wide 20%:

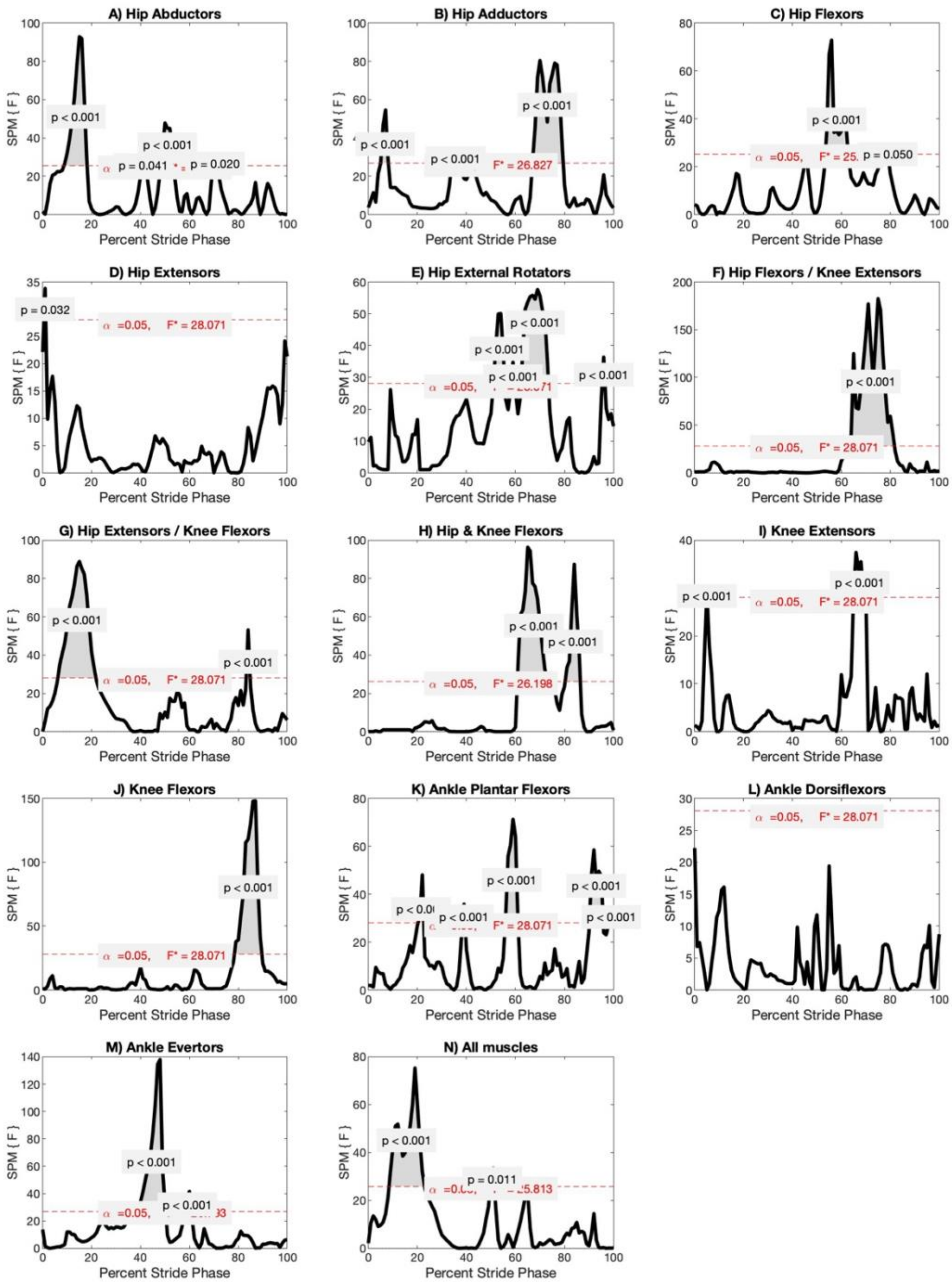

Biology Open • Supplementary information

Muscle metabolic power, Narrow 20%:

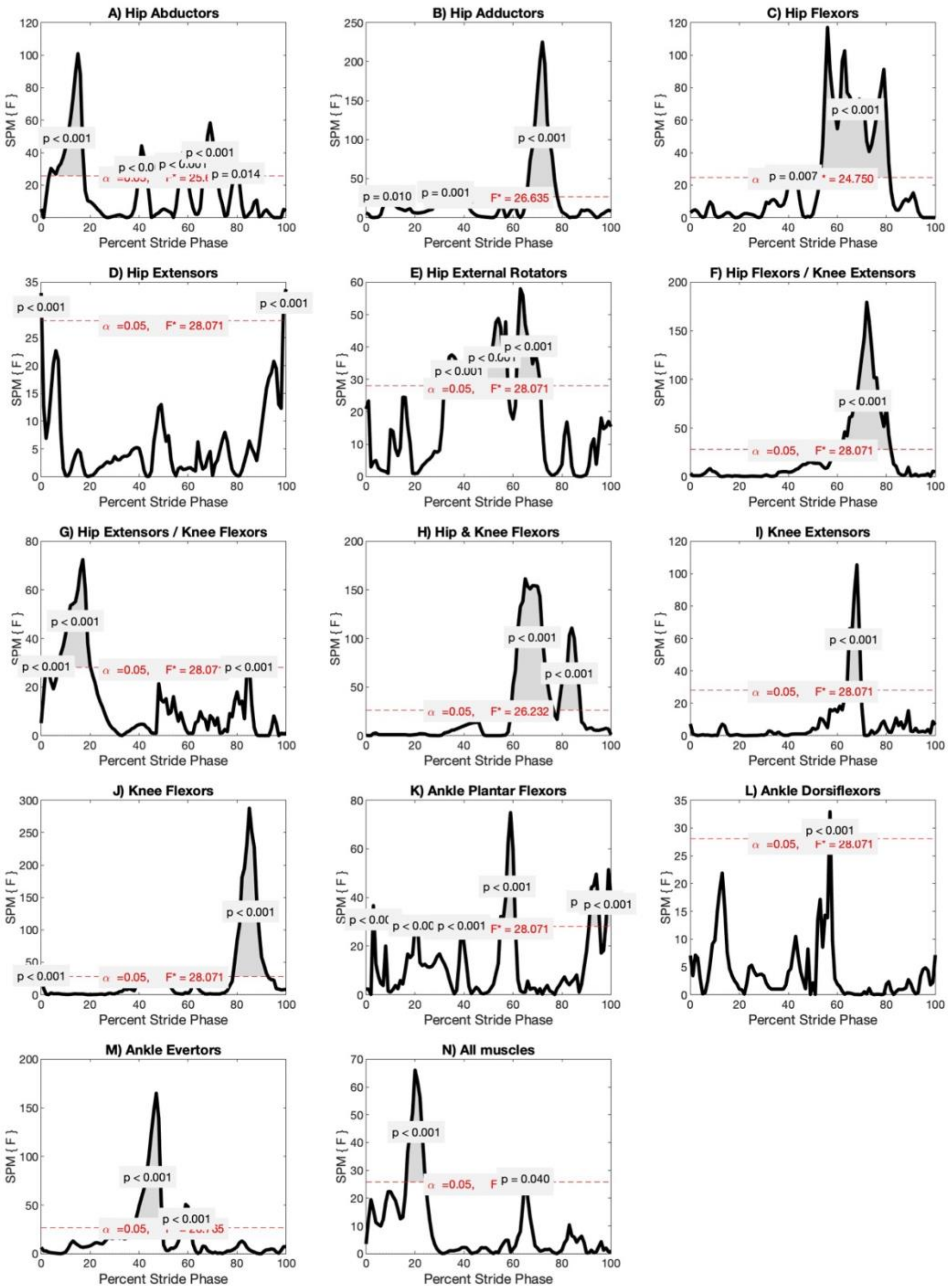

Biology Open • Supplementary information

Muscle metabolic power, Narrow 40%:

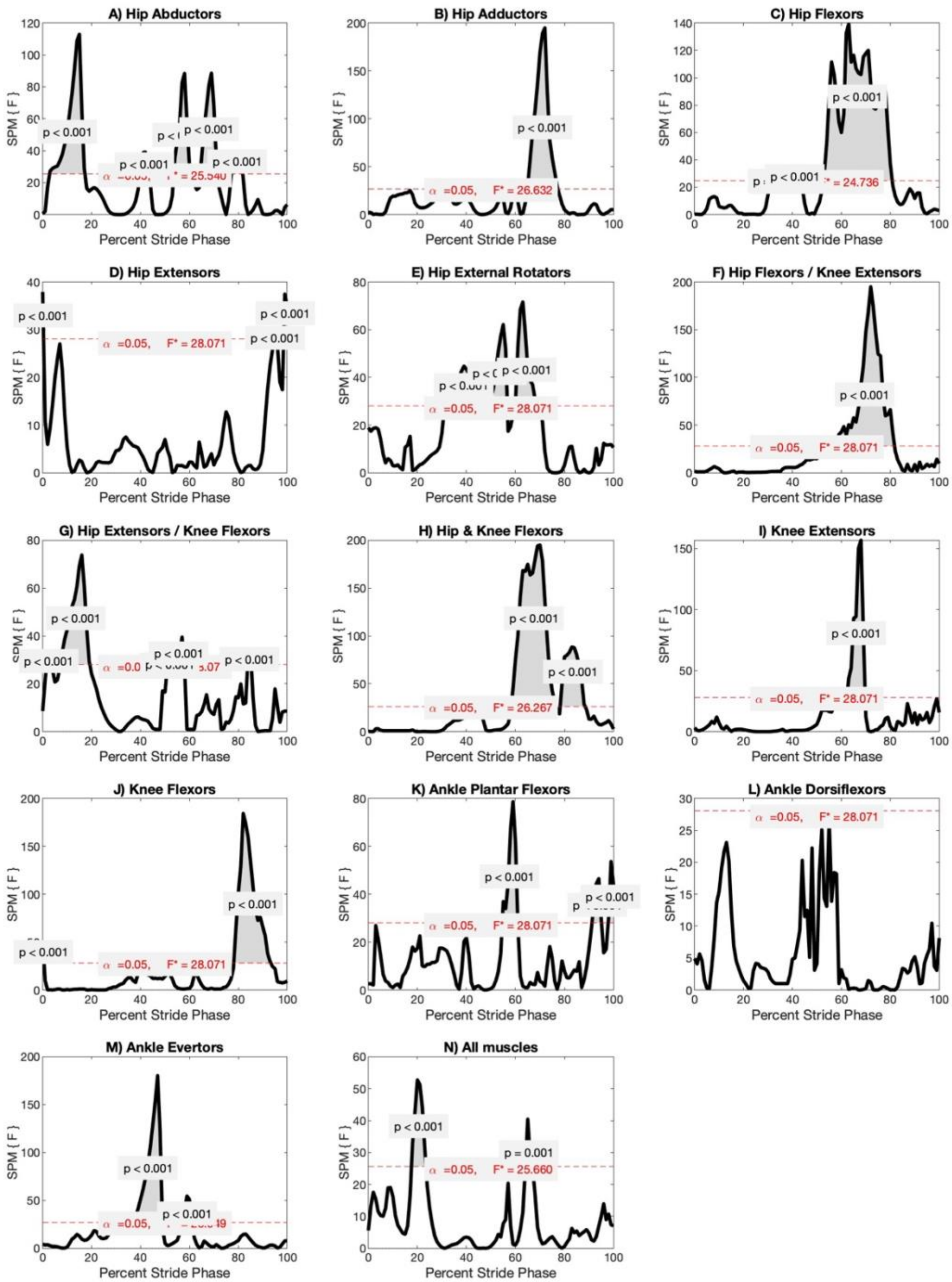

Fig. S2. Results from SPM analysis. Each figure demonstrates the results for a variable (muscle force magnitudes or muscle metabolic power estimate) and a comparison between the original pelvic segment's functional hip width and one of the four experimental conditions (+/-20% and 40%). In each subfigure, the black line is the F statistic and statistical significance is indicated with grey shading for $p<0.05$. These plots to no indicate the direction of any significant difference. Direction of any difference can be discerned from the previous figures.

Biology Open • Supplementary information

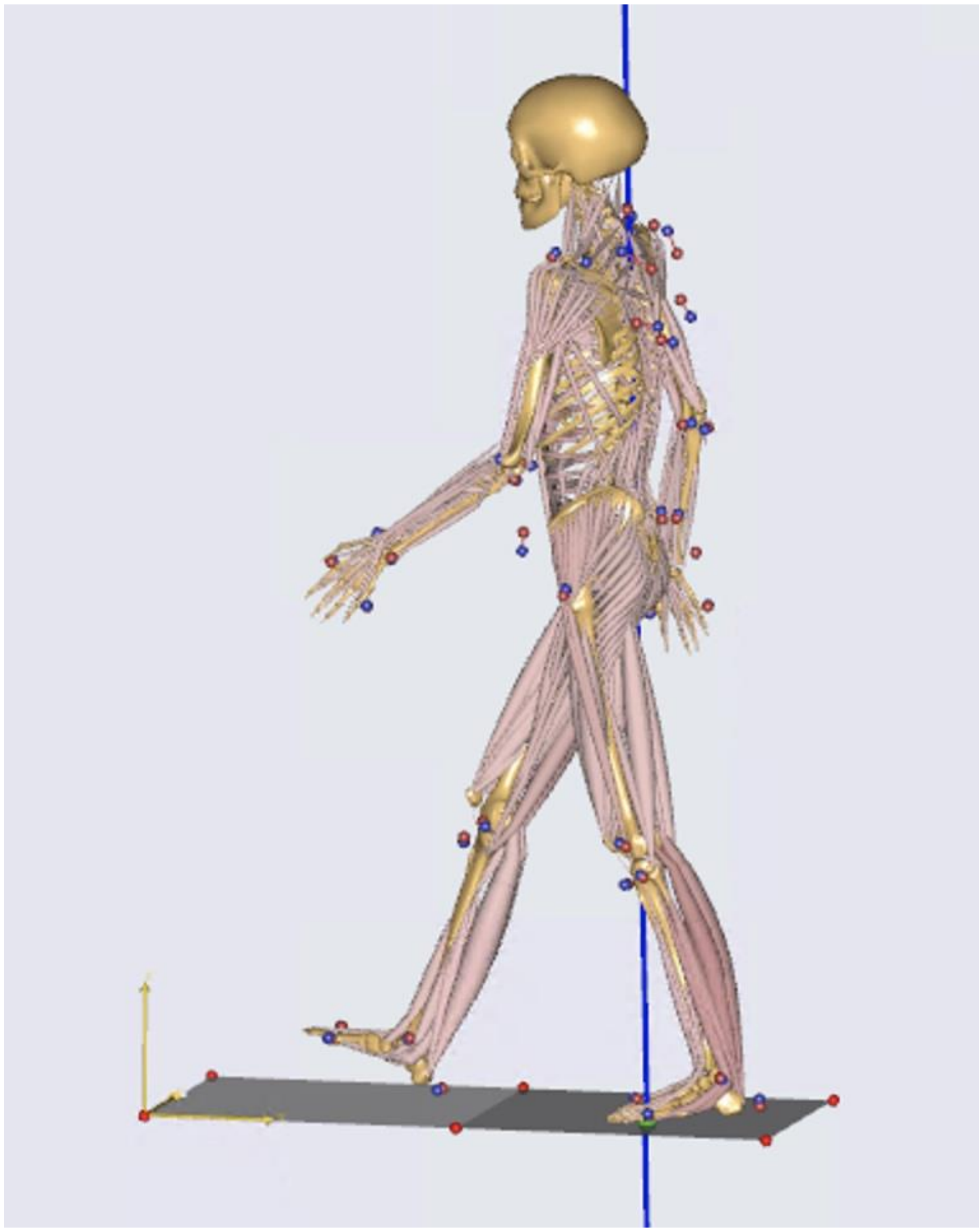

Fig. S3. AnyBody model (subject 2014019) just before contralateral heel strike (~ 50% of stride) with original, subject-size-scaled segments. In this trial, the left foot is the first stance foot and the subject is walking in the negative x-axis (denoted with yellow arrows) direction. Experimental markers are shown as blue spheres while marker drivers are red spheres. Muscle elements are indicated with pink volumes. Darker pink indicates higher muscle force (or activation). Note that the ankle plantar flexors of the stance foot are darker pink. The force plates are shown as grey rectangles with the active one darker gray. The resultant ground reaction force is the blue line. Bones are visualized in golden brown.
